# Field Site-Specific Effects of an *Azospirillum* Seed Inoculant on Key Microbial Functional Groups in the Rhizosphere

**DOI:** 10.3389/fmicb.2021.760512

**Published:** 2022-01-26

**Authors:** Sébastien Renoud, Jordan Vacheron, Danis Abrouk, Claire Prigent-Combaret, Laurent Legendre, Daniel Muller, Yvan Moënne-Loccoz

**Affiliations:** ^1^Univ Lyon, Université Claude Bernard Lyon 1, CNRS, INRAe, VetAgro Sup, UMR 5557 Ecologie Microbienne, Villeurbanne, France; ^2^Univ Lyon, Université de St Etienne, St Etienne, France

**Keywords:** microbial functional group, PGPR, inoculation, nitrogen fixers, ACC deaminase producers, 4-diacetylphloroglucinol producers

## Abstract

The beneficial effects of plant growth–promoting Rhizobacteria (PGPR) entail several interaction mechanisms with the plant or with other root-associated microorganisms. These microbial functions are carried out by multiple taxa within functional groups and contribute to rhizosphere functioning. It is likely that the inoculation of additional PGPR cells will modify the ecology of these functional groups. We also hypothesized that the inoculation effects on functional groups are site specific, similarly as the PGPR phytostimulation effects themselves. To test this, we assessed in the rhizosphere of field-grown maize the effect of seed inoculation with the phytostimulatory PGPR *Azospirillum lipoferum* CRT1 on the size and/or diversity of selected microbial functional groups important for plant growth, using quantitative polymerase chain reaction and/or Illumina MiSeq metabarcoding. The functional groups included bacteria able to fix nitrogen (a key nutrient for plant growth), producers of 1-aminocyclopropane-1-carboxylate (ACC) deaminase (which modulate ethylene metabolism in plant and stimulate root growth), and producers of 2,4-diacetylphloroglucinol (an auxinic signal enhancing root branching). To test the hypothesis that such ecological effects were site-specific, the functional groups were monitored at three different field sites, with four sampling times over two consecutive years. Despite poor inoculant survival, inoculation enhanced maize growth. It also increased the size of functional groups in the three field sites, at the maize six-leaf and flowering stages for diazotrophs and only at flowering stage for ACC deaminase and 2,4-diacetylphloroglucinol producers. Sequencing done in the second year revealed that inoculation modified the composition of diazotrophs (and of the total bacterial community) and to a lesser extent of ACC deaminase producers. This study revealed an ecological impact that was field specific (even though a few taxa were impacted in all fields) and of unexpected magnitude with the phytostimulatory *Azospirillum* inoculant, when considering microbial functional groups. Further methodological developments are needed to monitor additional functional groups important for soil functioning and plant growth under optimal or stress conditions.

## Introduction

Plant growth–promoting Rhizobacteria (PGPR) benefit plants mainly by (i) stimulating root system development, thereby allowing seedlings to explore larger soil volumes to gain access to water and mineral nutrients (Vacheron et al., 2013); (ii) enhancing nutrient availability, e.g., via N_2_ reduction or P solubilization (Dobbelaere et al., 2003; Basu et al., 2021); (iii) improving root system functioning in terms of ion uptake, by stimulating nutrient transporter expressions and/or activities in roots (Bertrand et al., 2000; Vacheron et al., 2013; Pii et al., 2015); and/or (iv) controlling root parasites via competition or antagonism, which leads to parasite inhibition (Raaijmakers et al., 2009; Basu et al., 2021). Effective PGPR strains have increased crop yields in many (but not all) field trials (Okon and Labandera-Gonzalez, 1994; El Zemrany et al., 2006; García de Salamone et al., 2010). Their use as crop inoculants is promising to reduce chemical inputs and improve farming sustainability, despite plant growth-promotion effects that can fluctuate according to the field, the year, or other farming/environmental parameters (Okon and Labandera-Gonzalez, 1994; Dobbelaere et al., 2003; Castro-Sowinski et al., 2007; Raaijmakers et al., 2009; Rozier et al., 2017; Basu et al., 2021).

*Azospirillum* is an emblematic PGPR genus and is widely used in certain countries to stimulate maize, wheat, and rice (Castro-Sowinski et al., 2007; Rozier et al., 2017; Schmidt and Gaudin, 2018). The main mode of action is the secretion of phytohormones especially indole acetic acid, involved in stimulation of root branching and growth (Steenhoudt and Vanderleyden, 2000). In certain *Azospirillum* strains, plant hormonal effects may also take place via synthesis of root-branching signal nitric oxide (Creus et al., 2005; Molina-Favero et al., 2008) or deamination of 1-aminocyclopropane-1-carboxylate (ACC), the ethylene precursor in plants (Vacheron et al., 2013; Glick, 2014). Associative nitrogen fixation also occurs in *Azospirillum*, although its contribution is considered minor (Steenhoudt and Vanderleyden, 2000).

The interaction between *Azospirillum* and plant leads to major changes in the physiology of both partners. On the bacterial side, more than 400 genes of *Azospirillum lipoferum* 4B were differentially expressed when the bacterium was in contact with the host plant rather than in the absence of the host (Drogue et al., 2014a). They were involved especially in detoxification of reactive oxygen species and multidrug efflux, which could be important for root colonization. On the plant side, *Azospirillum* resulted in modified expression of thousands of genes, including many involved in plant defense or ethylene/auxin pathways (Drogue et al., 2014b). Therefore, an *Azospirillum* inoculant is likely to change the nature or amount of compounds released by roots (i.e., rhizodeposition patterns), and indeed inoculation resulted in physiological changes in terms of rice content in secondary metabolites (Chamam et al., 2013); metabolite profiles of roots, shoots (Brusamarello-Santos et al., 2017), and xylem sap (Rozier et al., 2016) in maize; protein accumulation in maize seedlings (Cangahuala-Inocente et al., 2013); and production of flavonoids by roots of rice (Chamam et al., 2013) and alfalfa (Volpin et al., 1996). These chemical changes may lead to modified ecological conditions for microorganisms residing in the rhizosphere. Therefore, *Azospirillum* inoculation can be expected to trigger a range of indirect effects on other root-colonizing microorganisms, in addition to direct competition effects with resident rhizosphere populations. However, maize inoculation with *A. lipoferum* CRT1 caused only modest (but statistically significant) changes at the scale of the whole rhizobacterial community (Baudoin et al., 2009), as typically found also for other *Azospirillum* inoculants (Herschkovitz et al., 2005; Lerner et al., 2006; Felici et al., 2008; Naiman et al., 2009; Baudoin et al., 2010; García de Salamone et al., 2012; Bao et al., 2013; da Costa et al., 2018; Di Salvo et al., 2018). This may seem surprising considering the pronounced effects of *Azospirillum* on plant physiology, but perhaps methodological limitations account for these observations. Both direct and indirect microbial interactions between PGPR inoculants and the indigenous microbiota may, in turn, have an impact on rhizosphere functioning (Trabelsi and Mhamdi, 2013; Florio et al., 2017; Di Salvo et al., 2018; Dal Cortivo et al., 2020; Kusstatscher et al., 2020), but this possibility remains poorly documented as microbial functional groups have been neglected. In light of the low reproducibility of plant-beneficial effects of PGPR inoculants in field situations, there is a need to develop our knowledge on the ecological consequences of such inoculations. It is also important because indirect PGPR effects, that is, effects taking place via interactions with indigenous microorganisms, are increasingly considered important for phytostimulation (Trabelsi and Mhamdi, 2013; Vacheron et al., 2013; Ambrosini et al., 2016; Di Salvo et al., 2018; Kusstatscher et al., 2020). In other words, it might be that the low reproducibility of phytostimulation performance in fields could result, in part, from a low reproducibility of the interactions between microbial inoculants and resident microorganisms. To explore this possibility, a prerequisite is to understand the variability of PGPR effects on the indigenous microbiota, as the latter displays spatiotemporal heterogeneity in field conditions (Baudoin et al., 2009; Raaijmakers et al., 2009; García de Salamone et al., 2010; Schmidt and Gaudin, 2018; Kusstatscher et al., 2020; Renoud et al., 2020).

Microbial functioning of the rhizosphere involves a broad range of particular ecological functions carried out by functional groups, for example, nitrogen fixers, phytohormone producers, and so on. Often, each functional group comprised multiple taxa, which may contribute differently to the corresponding ecological function. In addition, the effects of environmental conditions (climate, soil type, plant genotype and phenology, etc.) are likely to differ from one functional group to the other (Fuhrman, 2009; Reed et al., 2011; Schimel and Schaeffer, 2012; Renoud et al., 2020). Therefore, it is not feasible to infer the impact of PGPR inoculation on rhizosphere-relevant microbial functional groups based solely on our knowledge of inoculant impact on the taxonomic composition of the rhizomicrobial community (Trabelsi and Mhamdi, 2013; Ambrosini et al., 2016). Here, we tested the hypotheses that (i) PGPR inoculation can lead to major changes in root-associated functional microbial communities important for plant development/growth, and (ii) these PGPR effects on functional communities may differ in a field site-specific manner.

To address this issue, we assessed the effect of maize seed inoculation with *A. lipoferum* CRT1 on the size (i.e., the number of microbial cells) and/or diversity (i.e., richness, genetic structure, and composition) of three microbial functional groups, that is, nitrogen fixers (using *nifH* marker), ACC deaminase producers (*acdS* marker), and producers of 2,4-diacetylphloroglucinol, a root-branching signal (Brazelton et al., 2008) that may also act as an antimicrobial compound if present at high concentration (Keel et al., 1990) (*phlD* marker). These functional groups were selected based on (i) their importance for phytostimulation (Dobbelaere et al., 2003; Brazelton et al., 2008; Vacheron et al., 2013; Schmidt and Gaudin, 2018); for example, N is a key nutrient for plant, and therefore, N fixation is a key aspect of rhizosphere functioning; (ii) the availability of knowledge on their mode of action and corresponding regulatory processes (Keel et al., 1990; Steenhoudt and Vanderleyden, 2000; Dobbelaere et al., 2003; Brazelton et al., 2008; Vacheron et al., 2013; Glick, 2014; Rozier et al., 2017); (iii) the possibility to monitor them by focusing on a single gene marker shared by all members of the functional group, that is, *nifH*, *acdS*, and *phlD*, respectively (Vacheron et al., 2013; Renoud et al., 2020); (iv) the possibility to infer taxonomic affiliations of individual functional group members from marker gene sequences (for nitrogen fixers and ACC deaminase producers) (Renoud et al., 2020); and (v) the availability of validated polymerase chain reaction (PCR) methodology to enable monitoring in soil systems (Poly et al., 2001; Bouffaud et al., 2016, 2018; Renoud et al., 2020). For instance, auxin synthesis and phosphate solubilization were not considered despite their ecological significance, as they rely on several different pathways, some of them probably not yet documented, which restricts the possibilities of monitoring. It is important to note that the inoculant used here (i.e., *A. lipoferum* CRT1) belongs to nitrogen fixers but not to ACC deaminase producers or 2,4-diacetylphloroglucinol producers.

To this end, the PGPR *A. lipoferum* CRT1 was used as maize seed inoculant in two consecutive years, at three field sites located in the region of Lyon, France, and corresponding to a luvisol (termed L; near Chatonnay), a fluvic cambisol (FC; near Sérézin-de-la-Tour), and a calcisol (C; near Saint Savin), and its impact was investigated by quantitative PCR (qPCR) and/or Illumina MiSeq-based metabarcoding. In addition, maize was grown under optimized or suboptimal conditions of mineral N fertilization to assess the significance of N limitation (nutriment stress) as a factor possibly modulating inoculation effects, based on measurements of the size of the three microbial functional groups. Monitoring was carried out at the six-leaf stage of growth (i.e., six leaves are produced, and maize now relies on its permanent root system rather than the seminal roots) and at flowering time, two key stages to assess maize functioning and performance (Gabriel et al., 2017; Sarabia et al., 2018).

## Materials and Methods

### Field Trials

The field experiments were run in 2014 and 2015 in Chatonnay (L), Sérézin-de-la-Tour (FC), and Saint Savin (C), located near Bourgoin-Jallieu (France). The soils are a luvisol (L), a fluvic cambisol (FC), and a calcisol (C) based on FAO soil classification, and chemical features are given in Supplementary Table 1.

These field experiments have been described (Rozier et al., 2017). At each location, the crop rotation includes 1 year of wheat (grown prior to the 2014 trial), 6 years of maize (starting with the 2014 and 2015 trials), and 1 year of rapeseed. Seeds of maize (*Zea mays* cv. Seiddi; provided by the Dauphinoise coop company, France) were sown on April 18 (at FC) and 23 (at C and L) in 2014 and on April 30 (C) and May 11 (FC and L) in 2015. The fields were not irrigated.

Three levels of mineral nitrogen fertilization were considered, that is, X (optimized N fertilization in one application carried out at the six-leaf stage), XS (the same N fertilization level but split into two applications), and 0 (no fertilizer). In 2014, the effect of inoculation with *A. lipoferum* CRT1 was studied at each of the three levels of mineral nitrogen fertilization (X, XS, and 0) for site FC and two levels (X and 0) for sites L and C, using a factorial design with, respectively, six or four combinations of factors (i.e., inoculation or no inoculation × three or two mineral N levels). The optimal dose X for maize (based on local agronomic assessments) was 120 kg mineral N/ha for sites FC and C and 180 kg mineral N/ha for site L. On site FC, the XS nitrogen treatment corresponded to the X fertilizer dose applied half at sowing and half on May 21 (at the six-leaf stage). Individual replicate plots were 12 (FC and C) or 8 (L) maize rows wide and 12 m long. They were organized along a randomized block design with five blocks. Mineral nitrogen fertilizer was applied on May 21 (FC and C) and 22 (L) for fertilized plots.

In 2015, CRT1 inoculation was studied at two N levels (X and 0). Inoculation (or not) and mineral N level were applied to the same plots that had received these applications the year before. In 2015, mineral nitrogen fertilizer was applied on June 5 at C and June 9 at FC and L (at the six-leaf stage).

### Inoculum Preparation and Inoculant Monitoring

*A. lipoferum* CRT1 was isolated in France from the rhizosphere of field-grown maize (Fages and Mulard, 1988) and promoted maize growth when used as inoculant (Fages and Mulard, 1988; Jacoud et al., 1999; Walker et al., 2011; Rozier et al., 2017, 2019). For inoculation, maize seeds were mixed with CRT1 cells included in a sterile peat-based formulation supplied by Agrauxine (Lesaffre Plant care; Angers, France), and the exact same peat-based seed formulation (but without any CRT1 cell added) was used in the non-inoculated control.

Inoculant survival in the rhizosphere was assessed by qPCR, using CRT1-specific primers Q1/Q2 (Couillerot et al., 2010). For quantification on seeds at sowing, inoculum level was also estimated by colony counts on nitrogen-free agar containing 0.2 g/L ammonium chloride and Congo red (Cáceres, 1982). At sowing, the inoculant was recovered in 2014 at 2.0 × 10^2^ colony-forming units (CFUs) (and equivalent to 6.0 × 10^3^ cells by qPCR) per seed, and in 2015 at 3.7 × 10^2^ CFUs (and equivalent to 3.0 × 10^4^ cells) per seed at sites L and FC, and 8.8 × 10^2^ CFUs (and equivalent to 1.5 × 10^5^ cells) per seed at site C.

### Sampling of Plants and DNA Extractions

In both years, maize was sampled at six-leaf and flowering stages, as described (Renoud et al., 2020). In 2014, all plots were studied. The six-leaf stage was sampled on May 25 (FC) and 26 (C and L), shortly after fertilizer application. On each plot, six plants were randomly chosen; their entire root system was dug up and shaken vigorously to dislodge loosely adhering soil. At sites FC and C, one pooled sample (i.e., six-root system) was obtained per plot, which made five pooled samples per treatment. At site L, each root system was treated individually to consider variations from one plant to the other, which made 6 root system × 5 plots = 30 samples per treatment. The sampling at flowering stage was done on July 8 (FC and C) and 9 (L), and six plants were sampled per plot and treated individually, which made 30 samples per treatment.

The moderate levels of plant-to-plant variation in 2014 prompted us to reduce samplings to four root systems per plot in 2015. In 2015, six-leaf maize was sampled on May 27 (C), June 5 (FC), and June 8 (L), and only non-fertilized plots were studied because fertilizers had not been applied yet. The flowering stage sampling was done on July 15 (C), 16 (FC), and 17 (L), and all plots were studied. At each sampling, the four root systems sampled per plot were treated individually, which made 20 samples per treatment.

Each root system sample was flash-frozen on the field, using liquid nitrogen, and lyophilized at the laboratory (24 h at –50°C), as described (Renoud et al., 2020). Briefly, roots and their adhering soil (i.e., rhizosphere; approximately 1 mm from root surface) were separated using brushes, and root-adhering soil was stored at –80°C. DNA was extracted from the latter using the FastDNA SPIN kit (BIO 101 Inc., Carlsbad, CA, United States). A total of 500-mg (for the 2014 pooled samples from FC and C) or 300-mg samples (for the other samples) were transferred in Lysing Matrix E tubes, as well as 5 μL of the internal standard APA9 (at 10^9^ copies/mL, with primers AV1f/AV1r) in order to normalize the efficiency of DNA extraction between rhizosphere samples (Park and Crowley, 2005; Couillerot et al., 2010). After 1-h incubation at 4°C, DNA was extracted and eluted in 50 mL of sterile ultrapure water, according to the manufacturer’s instructions, and DNA concentration assessed using Picogreen (Thermo Fisher Scientific).

### Quantitative Polymerase Chain Reaction Analysis of Microbial Functional Groups

The number of genes *nifH*, *acdS*, and *phlD* in the rhizosphere was assessed by qPCR using, respectively, primers polF/polR (Poly et al., 2001; Gaby and Buckley, 2017), acdSF5/acdSR8 (Bouffaud et al., 2018), and B2BF/B2BR3 (Almario et al., 2013). The *nifH* primers polF/polR have been advocated for combined qPCR and diversity analyses (Bouffaud et al., 2016; Gaby and Buckley, 2017), whereas the *acdS* primers acdSF5/acdSR8 have been validated for analysis of true *acdS* genes (i.e., without amplifying related D-cystein desulfhydrase genes coding for other types of PLP-dependent enzymes; Bouffaud et al., 2018). Methods are described in Supplementary File 1 (see also Renoud et al., 2020 for some of the genes).

### Sequencing Analysis of *nifH*, *acdS*, and All Rhizobacteria

Sequencing was carried out using maize at the six-leaf stage (i.e., prior to N fertilizer applications) in 2015, as described (Renoud et al., 2020). Briefly, equimolar composite samples of four rhizosphere DNA extracts (from four plants) were used per plot, that is, 5 plots × 3 fields = 15 samples per treatment. Illumina MiSeq sequencing (paired-end reads; 2 × 300 bp for *nifH* and *rrs*, 2 × 125 bp for *acdS*) was performed by MR DNA laboratory (Shallowater, TX, United States).^1^

*nifH*, *acdS*, and *rrs* sequencings were done using, respectively, primers polF/polR, acdSF5/acdSR8, and 515/806 targeting the V4 variable region (Yang et al., 2016; Renoud et al., 2020). All forward primers carried a barcode. The 30-cycle PCR (five cycles implemented on PCR products) was performed using the HotStarTaq Plus Master Mix Kit (Qiagen, Valencia, CA, United States) with the following conditions: 94°C for 3 min, followed by 28 cycles of 94°C for 30 s, 53°C for 40 s, and 72°C for 1 min, and a final elongation step at 72°C for 5 min. PCR products were checked in 2% (wt/vol) agarose gel to verify amplification success and relative band intensity. For each gene, the amplicons of the 15 samples were multiplexed (in equal proportions based on their molecular weight and DNA concentrations) and subsequently purified using calibrated Ampure XP beads prior to preparing a DNA library following Illumina TruSeq DNA library preparation protocol. Reads have been deposited in the European Bioinformatics Institute (EBI) database under accession numbers PRJEB14346 (*nifH*), PRJEB14343 (*acdS*), PRJEB14347 (*rrs*).

Sequence data were processed using the analysis pipeline of MR DNA (Renoud et al., 2020). Briefly, sequences were depleted of barcodes, the sequences < 150 bp or with ambiguous base calls were removed, the remaining sequences denoised, operational taxonomic units (OTUs; arbitrarily defined at 3% divergence threshold for the three genes) generated, and chimeras removed. Final OTUs from the *nifH* and *rrs* sequencing were taxonomically classified using BLASTn against a curated database derived from Greengenes (DeSantis et al., 2006), RDPII,^2^ and NCBI.^3^ Final OTUs of the *acdS* sequencing were classified using an in-house curated *acdS* database (Bouffaud et al., 2018). Datasets without singletons (here, singleton sequences are those found a single time among the 30 sequenced samples from inoculated or control treatments) were used to generate rarefaction curves and diversity indices of Shannon (H) and Simpson (calculated using sequencing subsample data for which each sample had the same number of sequences). Some of the sequences were used previously to describe microbial diversity of non-inoculated maize (Renoud et al., 2020).

### Statistical Analyses

Quantitative PCR data were compared by two-factor analysis of variance (i.e., inoculation × fertilizer treatment) and Fisher least significant difference tests, using log-transformed data.

Comparisons of bacterial diversity were carried out by between-class analysis (BCA) (Dolédec and Chessel, 1987) with the ADE4 (Chessel et al., 2004; Dray et al., 2007) and ggplot2 packages for R. BCA is a robust alternative to linear discriminant analysis (Huberty, 1994) when the number of samples is small compared with the number of predictors. When the number of samples is low, and particularly when it is lower than the number of predictors, discriminant analysis cannot be used. In this case, BCA can be very useful and provides illustrative graphical displays of between-group differences (Thioulouse et al., 2012). The significance of BCA results was assessed using a Monte Carlo test with 10,000 permutations (null hypothesis: absence of difference between groups). Non-metric multidimensional scaling (NMDS) was also performed, using the Bray–Curtis distance and the vegan package for R.

Two analyses were carried out to assess the impact of inoculation on particular genera. First, the genera contributing most to treatment differentiation (independently of their abundance) were identified by BCA; the position in the heatmap indicates the statistical importance of these taxa in positioning samples on the BCA axis with and without inoculation, these taxa being classified according to their importance in contributing to the construction of the BCA axis. As a complementary approach, the genus composition of the bacterial community and functional groups were characterized to consider the relative abundance of the most prevalent taxa. Unless otherwise stated, statistical analyses were performed using R version 3.3.2 at *p* < 0.05.^4^

## Results

### Effects of Inoculation on Maize Under Field Conditions

The three field trials have been described before (Rozier et al., 2017). The seed inoculant *A. lipoferum* CRT1 was under the detection threshold (of 4.0 × 10^3^ cell equivalents per gram of rhizosphere soil) at the stages sampled in the current work, that is, when maize had produced six leaves and at flowering time, in all three field sites studied and in both years. Previous results from these three fields indicated that seed inoculation had resulted in significant changes in (i) maize morphological properties at the six-leaf stage (which, in 2015, took place mainly at site L; Table 1), (ii) plant photosynthetic efficiency except at site L in 2015, (iii) metabolome (studied only in 2015) of maize roots at sites L and C as well as shoots at sites L and FC (i.e., contents of 13 metabolites in roots and 28 metabolites in leaves were significantly affected, with glutamine content modified both in roots and shoot at site FC), and/or (iv) yield at site L (only in 2015, with a significant positive effect), site FC (with a positive effect in 2014 and a negative effect in 2015), and site C (with a significant negative effect in 2014 and a trend for a 16% yield increase in 2015 but that was not statistically significant at *p* < 0.05) (Rozier et al., 2017). Therefore, inoculation effects were statistically significant but fluctuated according to field and year, which provided suitable experimental conditions in this work to assess field-level variability of inoculation impact on the rhizosphere microbiome.

**TABLE 1:** Effect of *Azospirillum lipoferum* CRT1 on root and shoot parameters of maize at 6 leaves in 2015 (i.e., the sampling date at which metabarcoding was implemented) in field sites L, FC, and C.

	Site L		Site FC		Site C	
	NI	I	NI	I	NI	I
Maximum photochemical yield	0.74 ± 0.05	0.77 ± 0.04	**0.71 ± 0.02**	0.75 ± 0.04*	**0.72 ± 0.03**	0.79 ± 0.03*
Shoot weigh (g plant^–1^)	**0.34 ± 0.07**	0.43 ± 0.11*	0.52 ± 0.14	0.59 ± 0.17	0.34 ± 0.08	0.39 ± 0.07
Leaf length (cm plant^–1^)	13.10 ± 1.63	14.02 ± 1.92	20.74 ± 1.71	19.82 ± 2.13	17.94 ± 2.42	17.18 ± 1.80
Leaf width (cm plant^–1^)	**1.53 ± 0.15**	1.68 ± 0.12*	1.61 ± 0.11	1.64 ± 0.14	1.71 ± 0.13	1.71 ± 0.17
Stem diameter (mm)	**6.87 ± 0.73**	7.63 ± 0.78*	7.74 ± 1.07	8.04 ± 0.99	7.24 ± 0.60	7.06 ± 0.84
Root weight (g plant^–1^)	**0.20 ± 0.05**	0.24 ± 0.05*	**0.28 ± 0.05**	0.23 ± 0.05*	0.27 ± 0.05	0.25 ± 0.05
Total root length (cm plant^–1^)	**212 ± 56**	303 ± 86*	418 ± 109	441 ± 90	323 ± 86	321 ± 61
Total root surface (cm^2^ plant^–1^)	**51.5 ± 12.0**	73.3 ± 20.3*	115.4 ± 29.0	116.0 ± 28.2	89.8 ± 23.9	81.7 ± 17.3
Average roots diameter (mm)	0.79 ± 0.13	0.78 ± 0.10	0.88 ± 0.08	0.83 ± 0.08	**0.89 ± 0.07**	0.81 ± 0.08*
Number of roots	**413 ± 120**	789 ± 469*	570 ± 88	671 ± 250	804 ± 536	497 ± 139

NI and I correspond respectively to non-inoculated and inoculated conditions. Stem diameter was measured at root collar and foliar morphology parameters on leaf number five. Statistical analyses were carried out using ANOVA (P < 0.05), and statistical differences between inoculated and non-inoculated treatments are indicated by stars (*) and bold characters. The data were obtained from Rozier et al. (2017), and they show that inoculation improved 8 of 10 maize parameters at field site L, whereas it increased maximum photochemical yield and decreased root biomass at site FC, and increased maximum photochemical yield and decreased root diameter at site C.

### Impact of Inoculation on Numbers of *nifH*, *acdS*, and *phlD* Rhizobacteria in Each Field

At the six-leaf stage in 2014, differences in the size of the *nifH* group between inoculated and non-inoculated maize were not significant at sites FC and C. At site L, however, inoculation resulted in statistically higher number of *nifH* bacteria in non-fertilized plots (nutriment stress) but lower number in fertilized plots (Table 2). At the six-leaf stage in 2015, the number of *nifH* bacteria (studied in non-fertilized plots) was statistically lower at site C upon inoculation, but no difference was found at the two other sites. At flowering stage in 2014, the size of the *nifH* group was statistically higher for inoculated than non-inoculated maize at sites L (with or without X-level fertilization) and FC (with XS-level fertilization) but not at site C. At flowering stage in 2015, a significant increase in the number of *nifH* bacteria following inoculation was found again, at sites FC (this time in both fertilization treatments) and C (in non-fertilized plots) (Table 2).

**TABLE 2:** Effect of seed inoculation with *Azospirillum lipoferum* CRT1 on the size of the *nifH*, *acdS*, and *phlD* functional groups in the maize rhizosphere at 6-leaf stage and flowering at three field sites L, FC, and C in 2014 and 2015.

		Site L				Site FC						Site C			
		X fertilizer		0 fertilizer		X fertilizer		XS fertilizer		0 fertilizer		X fertilizer		XS fertilizer	
		NI	I	NI	I	NI	I	NI	I	NI	I	NI	I	NI	I
***nifH* rhizobacteria**															
	2014-at 6 leaves	**7.60 ± 0.23**	7.01 ± 0.38 *	**7.02 ± 0.39**	7.52 ± 0.18 *	7.41 ± 0.42	7.43 ± 0.50	7.36 ± 0.34	7.53 ± 0.27	7.54 ± 0.13	7.44 ± 0.25	7.77 ± 0.59	7.20 ± 0.68	7.81 ± 0.80	8.10 ± 0.86
	2014-at flowering	**8.39 ± 0.31**	8.60 ± 0.21 *	**8.32 ± 0.27**	8.62 ± 0.20 *	8.77 ± 0.29	8.69 ± 0.44	**8.56 ± 0.23**	8.87 ± 0.31 *	8.66 ± 0.21	8.59 ± 0.24	9.07 ± 0.34	9.19 ± 0.39	9.17 ± 0.27	9.19 ± 0.17
	2015-at 6 leaves			8.40 ± 8.40	8.27 ± 8.27					8.11 ± 8.11	8.42 ± 8.42			**8.97 ± 8.97**	8.63 ± 8.63 *
	2015-at flowering	8.71 ± 0.26	8.78 ± 0.39	8.61 ± 0.28	8.80 ± 0.41	**7.75 ± 0.46**	8.52 ± 0.21 *			**7.75 ± 0.24**	8.45 ± 0.31 *	8.29 ± 0.30	8.29 ± 0.24	**7.99 ± 0.26**	8.45 ± 0.34 *
***acdS* rhizobacteria**															
	2014-at 6 leaves	**8.38 ± 0.25**	8.00 ± 0.29 *	8.09 ± 0.29	8.27 ± 0.16	8.02 ± 0.25	7.83 ± 0.32	8.11 ± 0.20	8.11 ± 0.30	8.24 ± 0.17	7.98 ± 0.27	8.20 ± 0.46	7.63 ± 0.46	7.90 ± 0.55	7.87 ± 0.51
	2014-at flowering	**7.80 ± 0.32**	8.06 ± 0.22 *	**7.71 ± 0.42**	8.13 ± 0.20 *	7.91 ± 0.23	7.82 ± 0.18	7.82 ± 0.21	8.04 ± 0.28	**7.92 ± 0.19**	7.71 ± 0.24 *	**8.21 ± 0.20**	8.48 ± 0.15 *	8.24 ± 0.18	8.22 ± 0.21
	2015-at 6 leaves			7.37 ± 7.37	7.40 ± 7.40					7.25 ± 7.25	7.15 ± 7.15			7.45 ± 7.45	7.33 ± 7.33
	2015-at flowering	7.70 ± 0.24	7.78 ± 0.32	**7.64 ± 0.20**	7.88 ± 0.41 *	**6.59 ± 0.25**	7.50 ± 0.15 *			**6.56 ± 0.12**	7.39 ± 0.18 *	**7.00 ± 0.21**	7.12 ± 0.14 *	6.92 ± 0.23	6.97 ± 0.20
***phlD* rhizobacteria**															
	2014-at 6 leaves	6.25 ± 0.53	6.03 ± 0.67	5.91 ± 0.47	5.89 ± 0.57	6.05 ± 0.69	5.70 ± 0.59	6.70 ± 0.72	6.07 ± 0.71	6.19 ± 1.06	5.86 ± 0.35	ND	ND	ND	ND
	2014-at flowering	5.91 ± 0.70	5.98 ± 0.35	5.62 ± 0.74	6.34 ± 0.44	6.11 ± 0.94	5.94 ± 0.86	6.48 ± 0.55	6.19 ± 0.76	6.01 ± 0.62	6.12 ± 0.74	ND	ND	ND	ND
	2015-at 6 leaves			5.82 ± 0.58	5.82 ± 0.49					6.25 ± 0.63	5.82 ± 0.87			ND	ND
	2015-at flowering	ND	ND	ND	ND	**5.31 ± 0.58**	6.24 ± 0.93 *			**4.99 ± 0.76**	5.85 ± 0.76 *	ND	ND	ND	ND

X and 0 correspond respectively to optimal N fertilizer dose (delivered half at sowing and half at 6 leaves in treatment XS) and no N applied. NI and I correspond respectively to non-inoculated and inoculated conditions. The analysis was done using pooled samples of six roots systems (n = 5) at FC and C and individual root systems (n = 30) at L in 2014, and individual root systems (n = 20) at all three sites in 2015. Data are shown in decimal log units, using means ± standard errors. ND, not detected (under the detection threshold of 3.2 × 10^3^ phlD copies per gram of rhizosphere soil). Statistical differences between inoculated and non-inoculated treatments (ANOVA and Fisher’s LSD tests, P < 0.05) are indicated by a star (*) and bold characters.

At the six-leaf stage in 2014, the size of the *acdS* group was statistically higher for inoculated than non-inoculated maize at site L in fertilized plots, but not in non-fertilized plots or at the other two sites (Table 2). There was no difference at the six-leaf stage in 2015. At flowering stage in 2014, a significant increase in the number of *acdS* bacteria following inoculation was found at sites L (in both fertilization treatments) and FC (with XS-level fertilization), whereas a decrease was observed at site FC (in non-fertilized plots). At flowering stage in 2015, the size of the *acdS* group was statistically higher for inoculated than non-inoculated maize at sites L (in non-fertilized plots), FC (in both fertilization treatments), and C (in fertilized plots).

At the six-leaf stage, there was no difference in the size of the *phlD* group between inoculated and non-inoculated maize, regardless of the site or the year (Table 2). At flowering stage in 2014, a significant increase in the number of *phlD* bacteria following inoculation was found at site L in the absence of fertilization, but there was no difference at the other site studied (FC). At flowering stage in 2015, the size of the *phlD* group was statistically higher with inoculated than non-inoculated maize at site FC, in both fertilization treatments.

In summary, when considering the four samplings carried out over the 2 years of the study, statistically significant differences in the size of functional groups resulting from inoculation were found in 8 of 19 comparisons at site L (4 of 7 for *nifH*, 4 of 7 for *acdS*, 0 of 5 for *phlD*), 8 of 27 comparisons at FC (3 of 9 for *nifH*, 3 of 9 for *acdS*, 2 of 9 for *phlD*), and 4 of 14 comparisons at C (2 of 7 for *nifH*, 2 of 7 for *acdS*), depending on year, maize growth stage, and restricted N fertilization level. Within a given field site, some but not all of the differences observed for various functional groups took place for the same comparisons, that is, at the same combinations of sampling date × N fertilization level.

### Impact of Inoculation on Diversity of *nifH*, *acdS*, and All Rhizobacteria in Each Field

**FIGURE 1:**
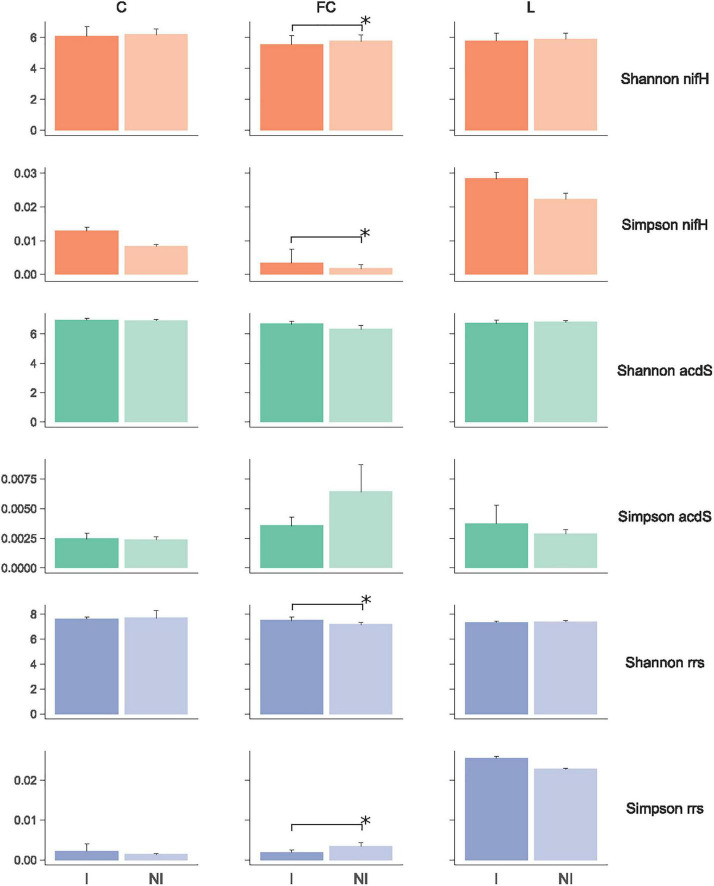
Diversity indices of *nifH* and *acdS* functional groups and the entire (*rrs*) bacterial community at sites L, FC, and C. NI and I correspond, respectively, to non-inoculated and inoculated conditions. Data are shown using means ± standard deviations (*n* = 5). Statistical analyses were carried out by Wilcoxon test between inoculated and non-inoculated conditions, and differences are shown with an asterisk (*p* < 0.05).

*nifH* sequencing of six-leaf maize rhizosphere (prior to N fertilizer applications) in 2015 gave 681,088 sequences, corresponding to 28,475 OTUs. The rarefaction curves reached a plateau (Supplementary Figure 1A), indicating that most of the *nifH* diversity had been recovered. Subsampling was done with 10,775 sequences per sample. There was no significant difference when comparing the resulting diversity indices between inoculated and non-inoculated maize, regardless of the index (Shannon or Simpson) and field site (Figure 1).

*acdS* sequencing resulted in 2,883,839 sequences, which gave 31,220 OTUs. Rarefaction analysis showed that the curves reached a plateau (Supplementary Figure 1B). Subsampling was implemented with 68,376 sequences per sample. At site FC, inoculated maize gave a significantly higher Shannon index (6.69 vs. 6.32; *p* = 0.032) but a lower Simpson index (3.6 × 10^–3^ vs. 6.4 × 10^–3^; *p* = 0.032) in comparison with non-inoculated maize. Inoculation had no effect on diversity level at the other sites (Figure 1).

*rrs* sequencing was also performed to determine whether inoculation effects could also take place at the scale of the whole rhizobacterial community. A total of 3,048,495 reads were obtained, corresponding to 38,419 OTUs. Rarefaction analysis indicated that curves reached a plateau (Supplementary Figure 1C). Subsampling was done with 51,696 sequences per sample. As for *acdS*, inoculated maize at site FC led for *rrs* data to a significantly higher Shannon index (7.52 vs. 7.19; *p* = 0.032) but a lower Simpson index (1.9 × 10^–3^ vs. 3.4 × 10^–3^; *p* = 0.046) in comparison with non-inoculated maize. Inoculation had no effect on diversity level at the other sites (Figure 1).

In summary, inoculation resulted in statistically significant differences in diversity indices for two of three metabarcoding comparisons at site FC (i.e., for *acdS* and *rrs*) and none of the six other metabarcoding comparisons (at sites L and C).

### Impact of Inoculation on Taxa Composition in Each Field

**FIGURE 2:**
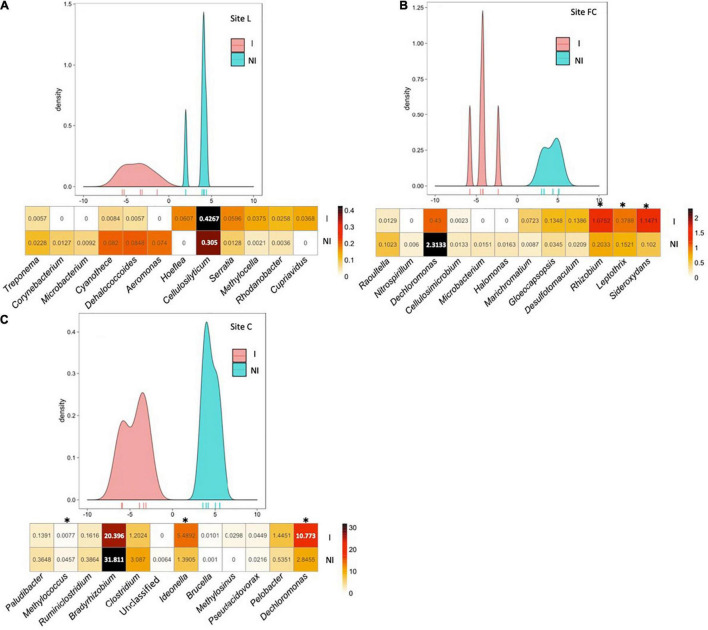
Between-class analysis of the effect of seed inoculation with *A. lipoferum* CRT1 on the composition of the *nifH* functional group in the maize rhizosphere at the six-leaf stage at three field sites L **(A)**, FC **(B)**, and C **(C)**, in 2015. NI and I correspond, respectively, to non-inoculated and inoculated conditions. For each site, the BCA graph is completed (below) by the distribution of the bacterial OTUs according to their contribution to treatment differentiation; BCA is supported statistically by a Monte Carlo test at sites L (*p* = 0.008), FC (*p* = 0.007), and C (*p* = 0.006), and the heatmap below indicates the average relative proportions for the 12 bacterial genera most impacted by inoculation in maize rhizosphere. Six bacterial genera most commonly associated with inoculation are indicated at the left, as well as six bacterial genera most often associated with the non-inoculated control (right). Statistical differences between inoculated and non-inoculated treatments are indicated by stars (*) (Wilcoxon test, *p* < 0.05). Heatmap unit is a percentage of the average relative proportion of taxa.

**FIGURE 3:**
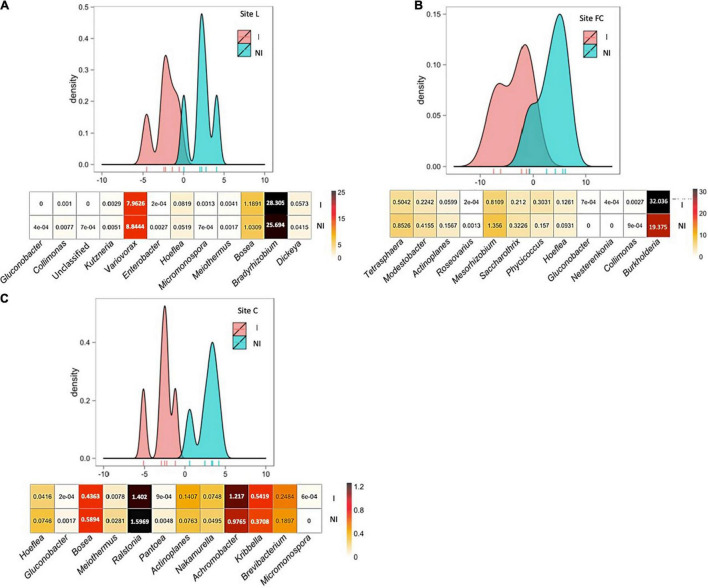
Between-class analysis of the effect of seed inoculation with *A. lipoferum* CRT1 on the composition of the *acdS* functional group in the maize rhizosphere at the six-leaf stage at three field sites L **(A)**, FC **(B)**, and C **(C)**, in 2015. NI and I correspond, respectively, to non-inoculated and inoculated conditions. For each site, the BCA graph is completed (below) by the distribution of the bacterial OTUs according to their contribution to treatment differentiation; BCA is statistically supported by a Monte Carlo test at sites L (*p* = 0.012), FC (*p* = 0.005), and C (*p* = 0.013), and the heatmap below indicates the average relative proportions for the 12 bacterial genera most impacted by inoculation in maize rhizosphere. Six bacterial genera most commonly associated with inoculation are indicated at the left, as well as six bacterial genera most often associated with the non-inoculated control (right). Differences between inoculated and non-inoculated treatments were not statistically significant (Wilcoxon test, *p* < 0.05). Heatmap unit is a percentage of the average relative proportion of taxa.

**FIGURE 4:**
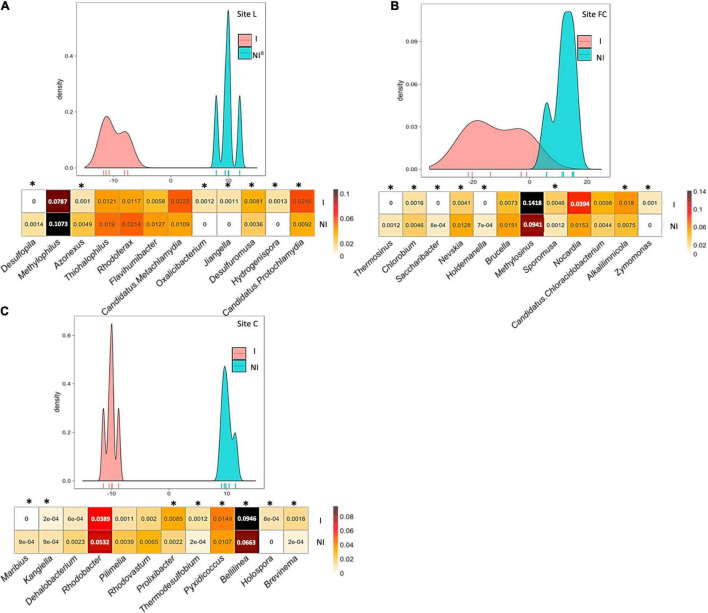
Between-class analysis of the effect of seed inoculation with *A. lipoferum* CRT1 on the composition of the total bacterial community in the maize rhizosphere at the six-leaf stage at three field sites L **(A)**, FC **(B)**, and C **(C)**, in 2015. NI and I correspond, respectively, to non-inoculated and inoculated conditions. For each site, the BCA graph is completed (below) by the distribution of the bacterial OTUs according to their contribution to treatment differentiation; BCA is statistically supported by a Monte Carlo test at sites L (*p* = 0.005), FC (*p* = 0.006), and C (*p* = 0.005), and the heatmap below indicates the relative proportions for the 12 bacterial genera most impacted by inoculation in maize rhizosphere. Six bacterial genera most commonly associated with inoculation are indicated at the left, as well as six bacterial genera most often associated with the non-inoculated control (right). Statistical differences between inoculated and non-inoculated treatments are indicated by stars (*) (Wilcoxon test, *p* < 0.05). Heatmap unit is a percentage of the average relative proportion of taxa.

NMDS evidenced that the main differences in the *nifH*, *acdS*, and *rrs* datasets of six-leaf maize were due to field site particularities, with inoculation impact apparent mainly at site FC based on *rrs* data (Supplementary Figure 2C). To unmask the overriding effects of field conditions, the impact of inoculation was assessed by BCA. Indeed, BCA of *nifH* sequences from 2015’s six-leaf maize indicated that the composition of the diazotroph community differed statistically at site L (*p* = 0.008), at site FC (*p* = 0.007), and at site C (*p* = 0.006) following inoculation with *A. lipoferum* CRT1 (Figures 2A–C). Similarly, BCA of *acdS* sequences from 2015’s six-leaf maize showed that the composition of ACC-deaminating rhizobacteria differed following inoculation with *A. lipoferum* CRT1, at sites L (*p* = 0.012), FC (*p* = 0.005), and C (*p* = 0.013) (Figures 3A–C). Finally, BCA of *rrs* sequences from 2015’s six-leaf maize indicated that the composition of rhizobacteria differed following inoculation with *A. lipoferum* CRT1, at sites L (*p* = 0.005), FC (*p* = 0.006), and C (*p* = 0.005) (Figures 4A–C).

BCA of *nifH* data showed that the six taxa most often associated with inoculation and the six taxa most commonly associated with the non-inoculated control displayed different rhizosphere levels overall between inoculated and non-inoculated maize. At site L, bacterial genera contributing most to the difference were (by decreasing order of importance) *Treponema*, *Corynebacterium*, *Microbacterium*, *Cyanothece*, *Dehalococcoides*, and *Aeromonas* (genera most commonly associated with inoculated maize), as well as *Hoeflea*, *Cellulosilyticum*, *Serratia*, *Methylocella*, *Rhodanobacter*, and *Cupriavidus* (genera most commonly associated with non-inoculated maize). At site FC, bacterial genera contributing most to the difference were *Raoultella*, *Nitrospirillum*, *Dechloromonas*, *Cellulosimicrobium*, *Microbacterium*, and *Halomonas* (genera most commonly associated with inoculated maize), as well as *Marichromatium*, *Gloeocapsopsis*, *Desulfomaculum*, *Rhizobium*, *Leptothrix*, and *Sideroxydans* (genera most commonly associated with non-inoculated maize). At site C, bacterial genera contributing most to the difference were *Paludibacter*, *Methylococcus*, *Ruminiclostridium*, *Bradyrhizobium*, *Clostridium* and an unclassified genus (genera most commonly associated with inoculated maize), as well as *Ideonella*, *Brucella*, *Methylosinus*, *Pseudacidovorax*, *Pelobacter*, and *Dechloromonas* (genera most commonly associated with non-inoculated maize).

BCA of *acdS* data indicated that the 12 most discriminating taxa displayed different rhizosphere levels overall between inoculated and non-inoculated maize at the sites. At site L, bacterial genera contributing most to the difference were *Gluconobacter*, *Collimonas*, an unclassified genus, *Kutzneria*, *Variovorax*, and *Enterobacter* (genera most commonly associated with inoculated maize); as well as *Hoeflea*, *Micromonospora*, *Meiothermus*, *Bosea*, *Bradyrhizobium*, and *Dickeya* (genera most commonly associated with non-inoculated maize). At site FC, bacterial genera contributing most to the difference were *Tetrasphaera*, *Modestobacter*, *Actinoplanes*, *Roseovarius*, *Mesorhizobium*, and *Saccharothrix* (genera most commonly associated with inoculated maize), as well as *Phycicoccus*, *Hoeflea*, *Gluconobacter*, *Nesterenkonia*, *Collimonas*, and *Burkholderia* (genera most commonly associated with non-inoculated maize). At site C, bacterial genera contributing most to the difference were *Hoeflea*, *Gluconobacter*, *Bosea*, *Meiothermus*, *Ralstonia*, and *Pantoea* (genera most commonly associated with inoculated maize), as well as *Actinoplanes*, *Nakamurella*, *Achromobacter*, *Kribbella*, *Brevibacterium*, and *Micromonospora* (genera most commonly associated with non-inoculated maize).

BCA of *rrs* data revealed that the 12 most discriminating taxa displayed different rhizosphere levels overall between inoculated and non-inoculated maize. At site L, bacterial genera contributing most to the difference were *Desulfopila*, *Methylophilus*, *Azonexus*, *Thiohalophilus*, *Rhodoferax*, and *Flavihumibacter* (genera most commonly associated with inoculated maize), as well as *Candidatus Metachlamydia*, *Oxalicibacterium*, *Jjiangella*, *Desulfuromusa*, *Hydrogenispora*, and *Candidatus Protochlamydia* (genera most commonly associated with non-inoculated maize). At site FC, bacterial genera contributing most to the difference were *Thermosinus*, *Chlorobium*, *Saccharibacter*, *Nevskia*, *Holdemanella*, and *Brucella* (genera most commonly associated with inoculated maize), as well as *Methylosinus*, *Sporomusa*, *Nocardia*, *Candidatus Chloracidobacterium*, *Alkalilimnicola*, and *Zymomonas* (genera most commonly associated with non-inoculated maize). At site C, bacterial genera contributing most to the difference were *Maribius*, *Kangiella*, *Dehalobacterium*, *Rhodobacter*, *Pilimelia*, and *Rhodovastum* (genera most commonly associated with inoculated maize), as well as *Prolixibacter*, *Thermodesulfobium*, *Pyxidicoccus*, *Bellilinea*, *Holospora*, and *Brevinema* (genera most commonly associated with non-inoculated maize).

Despite these rhizomicrobiota modifications, individual comparisons (Wilcoxon test) in the relative abundance of particular genera between inoculated and non-inoculated conditions did not show systematically a statistically significant change, due to low sample number (*n* = 5), high number of variables (e.g., *n* = 1,111 genera for the entire community; with the corresponding probability correction effect), and the fact that taxa found in only one of the two treatments were not necessarily found in all five replicates of that treatment. However, the comparison of genus composition data (Supplementary Figure 3) proved useful to emphasize trends among the most prevalent genera in functional groups (but not in the rhizobacterial community as a whole). Among the diazotrophs, there were trends for *Azospirillum*, *Bradyrhizobium*, *Geobacter* (at all three sites), *Desulfovibrio* (at sites L and FC), *Skermanella*, *Clostridium*, and *Rhizobium* (at site L) being more prevalent in inoculated maize, and for *Dechloromonas* (at all three sites), *Ruminiclostridium* (at sites L and FC), *Azoarcus*, *Pseudomonas*, *Leptothrix*, *Cellulosilyticum*, *Ideonella* (at site L), and *Sideroxydans* (at site FC) being less prevalent in inoculated maize. However, contrasted trends were observed for *Paenibacillus* (prevalence in inoculated maize higher at sites FC and C but lower at site L), *Pelosinus* (higher at L and C but lower at FC), and *Clostridium*, *Skermanella*, and *Rhizobium* (all three higher at L but lower at, respectively, FC, C, and both FC and C). Among the ACC deaminase producers, there was a trend for *Variovorax* (at site FC) being more prevalent in inoculated maize, and for *Burkholderia*, *Bradyrhizobium* (at site FC; thus, the trend for *acdS*^+^ *Bradyrhizobium* at FC was opposite to the trend for the entire genus; see above), and *Methylibium* (at site C) being less prevalent in inoculated maize.

In summary, inoculation with *A. lipoferum* CRT1 resulted in significantly different compositions of the diazotroph community, the ACC-deaminating community, and the global bacterial community, at each of the field sites L, FC, and C. For each of the three communities, the bacterial genera contributing most to the distinction between inoculated and non-inoculated maize differed from one field to the other (except that *Microbacterium* was more prevalent among diazotrophs in inoculated maize than the control at both sites L and FC), indicating field-specific inoculation effects on indigenous bacteria.

### Taxa Indicative of Inoculation Impact Across the Three Fields

**FIGURE 5:**
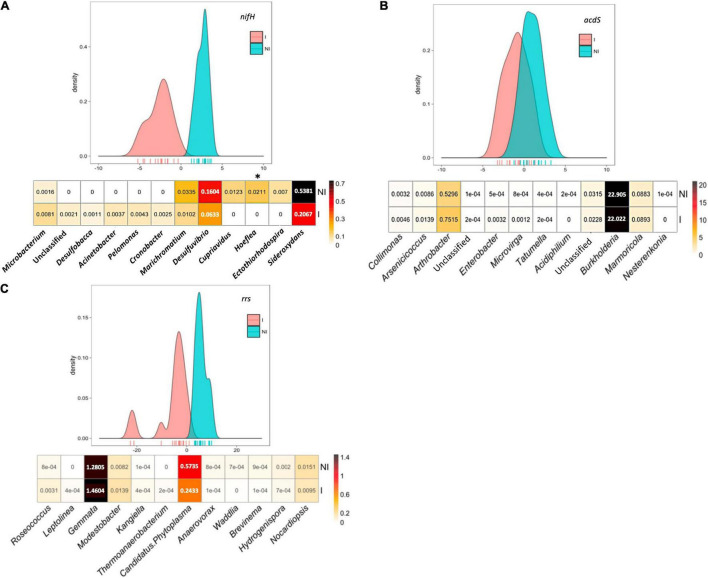
Between-class analysis of the effect of seed inoculation with *A. lipoferum* CRT1 on the composition of **(A)** *nifH* and **(B)** *acdS* functional groups, as well as **(C)** the total bacterial community (*rrs*) over three sites. Analyses were done on pooled data of bacterial composition of maize rhizosphere of the three sites studied. NI and I correspond, respectively, to non-inoculated and inoculated conditions. For each bacterial group, the BCA graph is completed (below) by the distribution of the bacterial OTUs according to their contribution to treatment differentiation. BCA is statistically supported by a Monte Carlo test based on *nifH* (*p* = 0.001) and *rrs* data (*p* = 10^– 4^), but not with *acdS* data (*p* = 0.19), and the heatmap below indicates the relative proportions for the 12 bacterial genera most impacted by inoculation in maize rhizosphere. Six bacterial genera most commonly associated with inoculation are indicated at the left, as well as six bacterial genera most often associated with the non-inoculated control (right). Statistical differences between inoculated and non-inoculated treatments are indicated by stars (*) (Wilcoxon test, *p* < 0.05). Heatmap unit is a percentage of the average relative proportion of taxa.

Even though the bacterial genera mainly implicated in functional group (and whole community) differences between inoculated maize and the control mostly differed from one field to the other, an attempt was made to identify differentiating taxa (including taxa with lower contributions) that would contribute to treatment differentiation more reproducibly, that is, across all three sites. To this aim, we pooled bacterial composition data from the maize rhizosphere of the three fields to determine which bacterial genera were the most impacted by *Azospirillum* inoculation at the scale of the three sites studied (Figures 5A–C). With this approach, inoculated and non-inoculated maize could be discriminated by BCA based on *nifH* (*p* = 0.001) or *rrs* data (*p* = 10^–4^), but not with *acdS* data (*p* = 0.19) despite apparent differences in the genus composition profile at two of three sites (Supplementary Figure 3).

With *nifH* data, the 12 bacterial genera contributing most to treatment differentiation were an unclassified genus, *Microbacterium*, *Desulfobacca*, *Acinetobacter*, *Pelomonas*, and *Cronobacter* (genera most commonly associated with inoculated maize), as well as *Marichromatium*, *Desulfurivibrio*, *Cupriavidus*, *Hoeflea*, *Ectothiorhodospira*, and *Sideroxydans* (genera most commonly associated with non-inoculated maize). With *rrs* data, the 12 genera contributing most to treatment differentiation were *Roseococcus*, *Leptolinea*, *Gemmata*, *Modestobacter*, *Kangella*, and *Thermoanaerobacterium* (genera most commonly associated with inoculated maize), as well as *Candidatus Phytoplasma*, *Anaerovorax*, *Waddlia*, *Brevinema*, *Hydrogenispora*, and *Nocardiopsis* (genera most commonly associated with non-inoculated maize).

In summary, in our search for more cosmopolitan taxa indicators of inoculation, the artificial pooling of metabarcoding sequences from the three sites did evidence additional taxa (on top of *Microbacterium*) among the 12 taxa contributing most to differentiation of inoculated maize from the non-inoculated control when considering the diazotroph community (and the global bacterial community), but not the ACC-deaminating community.

## Discussion

In this work, we tested the hypothesis that PGPR inoculation could result in major changes in root-associated functional microbial communities implicated in phytostimulation, using a range of field trials where the seed inoculant *A. lipoferum* CRT1 had triggered changes in maize metabolome, photosynthetic efficiency, plant development/growth, and/or crop yield (Rozier et al., 2017). Indeed, we observed a modification of strong magnitude in the composition of the diazotroph community and (to a lower degree) of the ACC deaminase community. It is interesting to note that, for both functional groups, these impacts took place irrespective of the morphological or physiological effects evidenced on maize plants at the time of sampling. Rice inoculation with *Azospirillum* (or *Pseudomonas*) in a field had little effect on *nifH* T-RFLP profiles (García de Salamone et al., 2012), whereas maize inoculation with nitrogen-fixing *Pseudomonas* in pots changed diazotroph community composition based on Illumina MiSeq sequencing of *nifH* (Ke et al., 2019). The issue was not investigated so far with the ACC deaminase community, as the methodology needed to do so has not been available for long (Bouffaud et al., 2018). The impact of inoculation on functional groups needs to receive further research attention, because it may bring additional insight into PGPR modes of action when the latter entail stimulation of indigenous plant-beneficial microorganisms (Trabelsi and Mhamdi, 2013; Vacheron et al., 2013; Ambrosini et al., 2016; da Costa et al., 2018) and also because it is likely to be more informative than taxonomic studies to describe the breadth of inoculant impacts (Ke et al., 2019).

Here, the significant effects of inoculation on the diversity of the *nifH* and *acdS* functional groups were also evidenced with *rrs* data, that is, when considering inoculation effects at the level of the whole rhizobacterial community. Based on *nifH*, *acdS*, and *rrs* analyses, the genera most impacted by inoculation belonged to *Actinobacteria*, *Cyanobacteria*, *Firmicutes*, or *Proteobacteria*, indicating a taxonomic shift larger than what was thought so far. Overall, the effects of inoculation were underpinned by changes in the relative abundance of multiple taxa. Some of these changes were small, but others were of larger magnitude (e.g., a decrease from 32 to 19% for *Burkholderia*; Figure 3). These findings contrast with previous studies showing minor and/or transient ecological impact of *Azospirillum* inoculants on the resident bacterial community colonizing the rhizosphere (Ambrosini et al., 2016), by molecular fingerprinting in the case of maize (Herschkovitz et al., 2005; Lerner et al., 2006; Matsumura et al., 2015), wheat (Naiman et al., 2009; Baudoin et al., 2010), rice (Pedraza et al., 2009; García de Salamone et al., 2010, 2012; Bao et al., 2013), or other crops (Correa et al., 2007; Felici et al., 2008), as well as by Illumina MiSeq metabarcoding in the case of maize (da Costa et al., 2018). Modest inoculant impacts on the rhizobacterial community were also found with *Bacillus* on lettuce (by metagenomics; Kröber et al., 2014) and tomato (by pyrosequencing; Qiao et al., 2017), *Pseudomonas* on lettuce (by DGGE and pyrosequencing; Schreiter et al., 2014), *Pseudomonas* or *Achromobacter* on maize (by Illumina MiSeq; da Costa et al., 2018), *Stenotrophomonas* on maize (by Illumina MiSeq; Kusstatscher et al., 2020), multispecies inoculants on tomato (by Illumina MiSeq; Nuzzo et al., 2020) and wheat (by pyrosequencing; Dal Cortivo et al., 2020), or a multispecies organic amendment on sugarcane (by Illumina MiSeq; Berg et al., 2019), whereas a larger impact was observed by Illumina MiSeq with *Pseudomonas* on maize (Ke et al., 2019) and different oilseed crops (Jiménez et al., 2020); *Pseudomonas*, *Paenibacillus*, or *Bacillus* on lettuce (Passera et al., 2020); an organic amendment enriched in microorganisms on strawberry (Deng et al., 2019); or a multispecies inoculant on onion (Pellegrini et al., 2021).

The second hypothesis tested was that the effects of PGPR inoculation on functional communities could be field site-specific, which turned out to be the case. Indeed, the most impacted bacterial genera seldom overlapped between sites, and when they did, modifications did not follow the same trend in all sites. Genus overlap (i.e., the same genera impacted at several sites) was more often found in the *acdS* group, perhaps because there were fewer genera in this group. When we pooled data from all fields, we showed that the *acdS* group did not differ significantly between inoculated and non-inoculated treatments, in contrary to the *nifH* group (and the *rrs* community). This suggests that inoculation effects on *acdS* rhizobacteria were more site-dependent than those on *nifH* rhizobacteria and on all rhizobacteria. These findings are likely to have implications in terms of rhizosphere functioning, phytostimulation implementation, and crop behavior, because they mean that inoculants will stimulate indigenous plant-beneficial microorganisms in a field-specific manner, as the natural soil community, the cropping/agronomic history, the pedoclimatic conditions, and/or crop physiology may differ between fields.

Here, inoculation resulted in 16 increases and 4 decreases in gene copy number (for *nifH*, *acdS*, or *phlD*) of the 60 individual comparisons carried out. These significant differences depended on the gene studied, the field, the year, the crop stage, and nitrogen fertilization (nutriment stress or not). Importantly, they often varied between fields when a given gene × nitrogen fertilization combination was compared across the various sampling dates assessed, pointing again to site-specific effects of inoculation on microbial functional groups important for phytostimulation.

As the *nifH*^+^ inoculant *A. lipoferum* CRT1 remained under qPCR detection threshold, it suggests that the augmentation in *nifH* copy number in CRT1-inoculated maize in certain comparisons was not due to presence of the *nifH*^+^ inoculant itself. Lack of inoculant survival does not necessarily mean that inoculation has no effect (Mawarda et al., 2020). Furthermore, inoculation effects on the density of the three functional groups were stronger at flowering stage, even though the inoculant was long below detection limit. Inoculation of *Ensifer* (previously *Sinorhizobium*) *meliloti* resulted also in changes in the number of *nifH*^+^ bacteria in maize rhizosphere that were plant phenology-dependent (Babić et al., 2008; Gupta et al., 2012). *acdS* gene copies were higher in CRT1-inoculated maize at flowering stage, which could have ecological consequences (Glick, 2014) as ethylene is produced from ACC in higher amount at flowering stage than in early growth stages (Bleecker and Kende, 2000).

Two types of mechanisms could explain the ecological impact of *A. lipoferum* CRT1: first, a direct effect of the inoculant on indigenous rhizobacteria by antagonism, competition, or cooperation (Pandey and Kumar, 1990; Tapia-Hernández et al., 1990; Tortora et al., 2011; Trabelsi and Mhamdi, 2013; Ambrosini et al., 2016), which could modify community composition. So far, *Azospirillum* species are mostly described as plant stimulators (Dobbelaere et al., 2003; Bashan and de-Bashan, 2010) by their capacity to produce phytohormones (Karadeniz et al., 2006; Cohen et al., 2015), but they may interact with other plant-beneficial microorganisms (Russo et al., 2005; Combes-Meynet et al., 2011). Here, the lack of significant inoculant survival (strain CRT1 remaining perhaps at very low levels, as a member of the rare biosphere) means that direct inoculant effects could occur only at the very early stages of maize colonization, where an impact on keystone “hub” microorganisms (Agler et al., 2016; Mawarda et al., 2020) might, in turn, have affected the microbiome network. Second, several studies showed that *A. lipoferum* CRT1 does not need to be well established in the maize rhizosphere to benefit plant growth (Jacoud et al., 1999; Rozier et al., 2017, 2019). In the current work, inoculation had resulted in changes in plant morphology and physiology (photosynthetic efficiency and metabolome), and at the six-leaf stage in 2015, plant growth modifications were observed at site L and plant metabolome changes at all three sites (Rozier et al., 2017). This starter effect of *A. lipoferum* CRT1 suggests modification of plant physiology from very early development stages of maize (Jacoud et al., 1999; Walker et al., 2011; Rozier et al., 2017, 2019), probably driving root microbiota changes. Indeed, CRT1 inoculation can also result in modification of plant secondary metabolites, especially benzoxazinoids and cinnamic acids (Walker et al., 2011), which are present in root secretions and known to play a major role in plant–microbe interactions (Neal et al., 2012).

## Conclusion

In conclusion, the seed inoculant *A. lipoferum* CRT1 did not manage to establish itself in the maize rhizosphere. However, it had a significant impact on the diversity of key functional groups important for plant performance and of the whole bacterial community in the rhizosphere, pointing to direct and/or plant-mediated effects of the inoculant on resident rhizobacteria. Importantly, we showed that this impact was field site-specific, thereby validating our hypothesis. This study demonstrated that inoculation has the potential to affect rhizosphere microbial functioning and identified community-based ecological mechanisms by which an inoculant could influence plant performance. It also showed the usefulness of considering microbial functional groups to reveal ecological effects on the plant microbiome, and further research efforts are necessary to develop the molecular toolbox needed to monitor a wider range of plant-beneficial functions under optimized or stress conditions.

## Data Availability Statement

The datasets presented in this study can be found in online repositories. The names of the repository/repositories and accession number(s) can be found in the article.

## Author Contributions

LL, YM-L, CP-C, and DM designed the project. SR, JV, CP-C, LL, YM-L, and DM carried out field work. SR and JV conducted the molecular work. SR and DA implemented bioinformatic analyses. SR, YM-L, and DM analyzed data and prepared the first draft of the manuscript, which was finalized by all authors.

## Conflict of Interest

The authors declare that the research was conducted in the absence of any commercial or financial relationships that could be construed as a potential conflict of interest.

## Publisher’s Note

All claims expressed in this article are solely those of the authors and do not necessarily represent those of their affiliated organizations, or those of the publisher, the editors and the reviewers. Any product that may be evaluated in this article, or claim that may be made by its manufacturer, is not guaranteed or endorsed by the publisher.

## Footnotes

1
www.mrdnalab.com

2
http://rdp.cme.msu.edu

3
www.ncbi.nlm.nih.gov

4
https://www.r-project.org/

## Supplementary Material

Supplementary Figure 1 Rarefaction curves for *nifH* **(A)**, *acdS* **(B)**, and *rrs* **(C)** at field sites L, FC, and C. NI and I correspond, respectively, to non-inoculated and inoculated conditions.

Supplementary Figure 2 Non-metric multidimensional scaling (NMDS) of *nifH* **(A)**, *acdS* **(B)**, and *rrs* **(C)** data at field sites L, FC, and C. NI and I correspond, respectively, to non-inoculated and inoculated conditions. NMDS of distance matrices was based on the Bray–Curtis distance and was done using the vegan package in R.

Supplementary Figure 3 Relative abundance of the most prevalent bacterial genera based on analysis of *nifH*, *acdS* and *rrs* data for field sites L, FC, and C (results from the five replicates were pooled). NI and I correspond, respectively, to non-inoculated and inoculated conditions.
