# Supplementary figures and images for: Field Site-Specific Effects of an Azospirillum Seed Inoculant on Key Microbial Functional Groups in the Rhizosphere
Source: Front Microbiol. 2022 Jan 26;12:760512. doi: 10.3389/fmicb.2021.760512 (PMC8825484; doi:10.3389/fmicb.2021.760512)

A

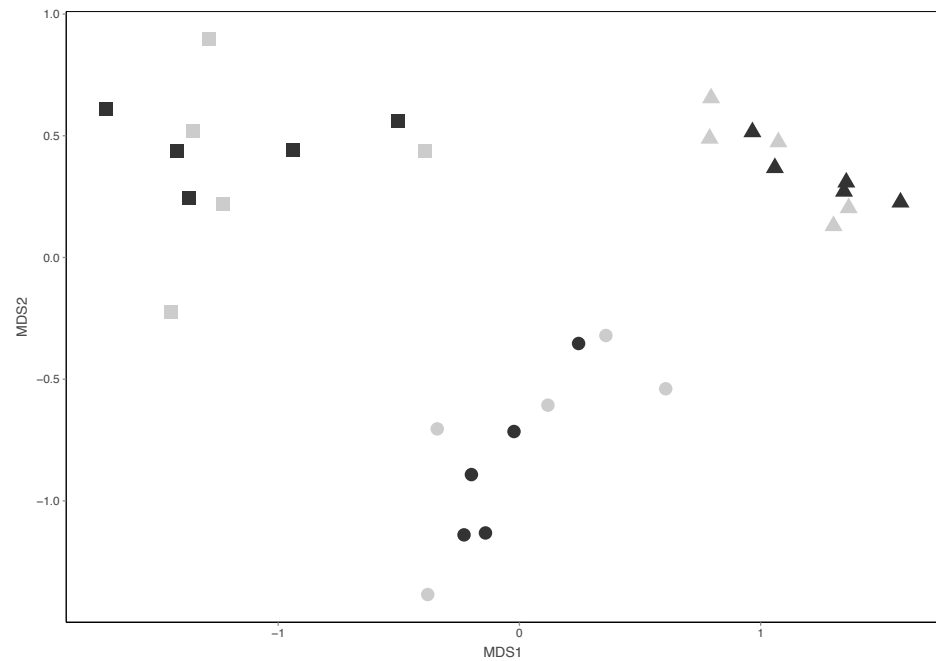

B

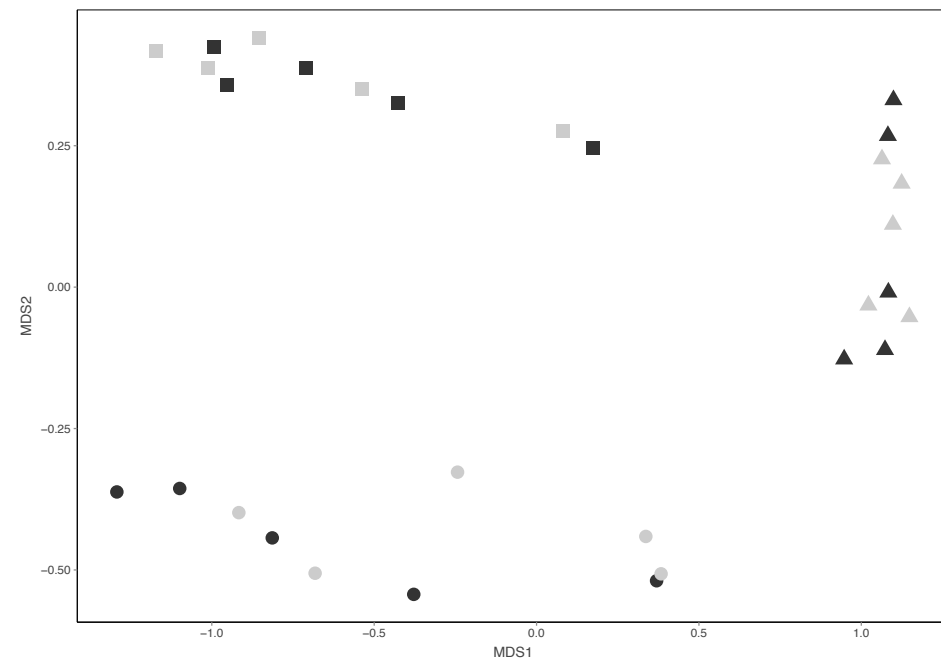

C

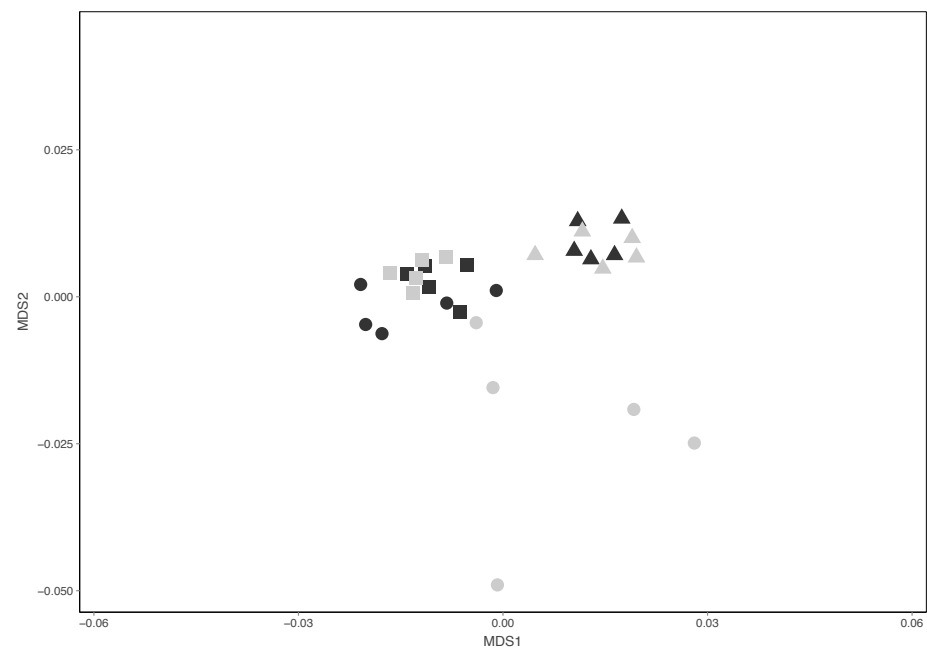

site

- FC
- C
- L

inoc

- NI
- I

Supplement: Supplementary Figure 1 — Rarefaction curves for nifH (A), acdS (B), and rrs (C) at field sites L, FC, and C. NI and I correspond, respectively, to non-inoculated and inoculated conditions. [file Data_Sheet_3.PDF]

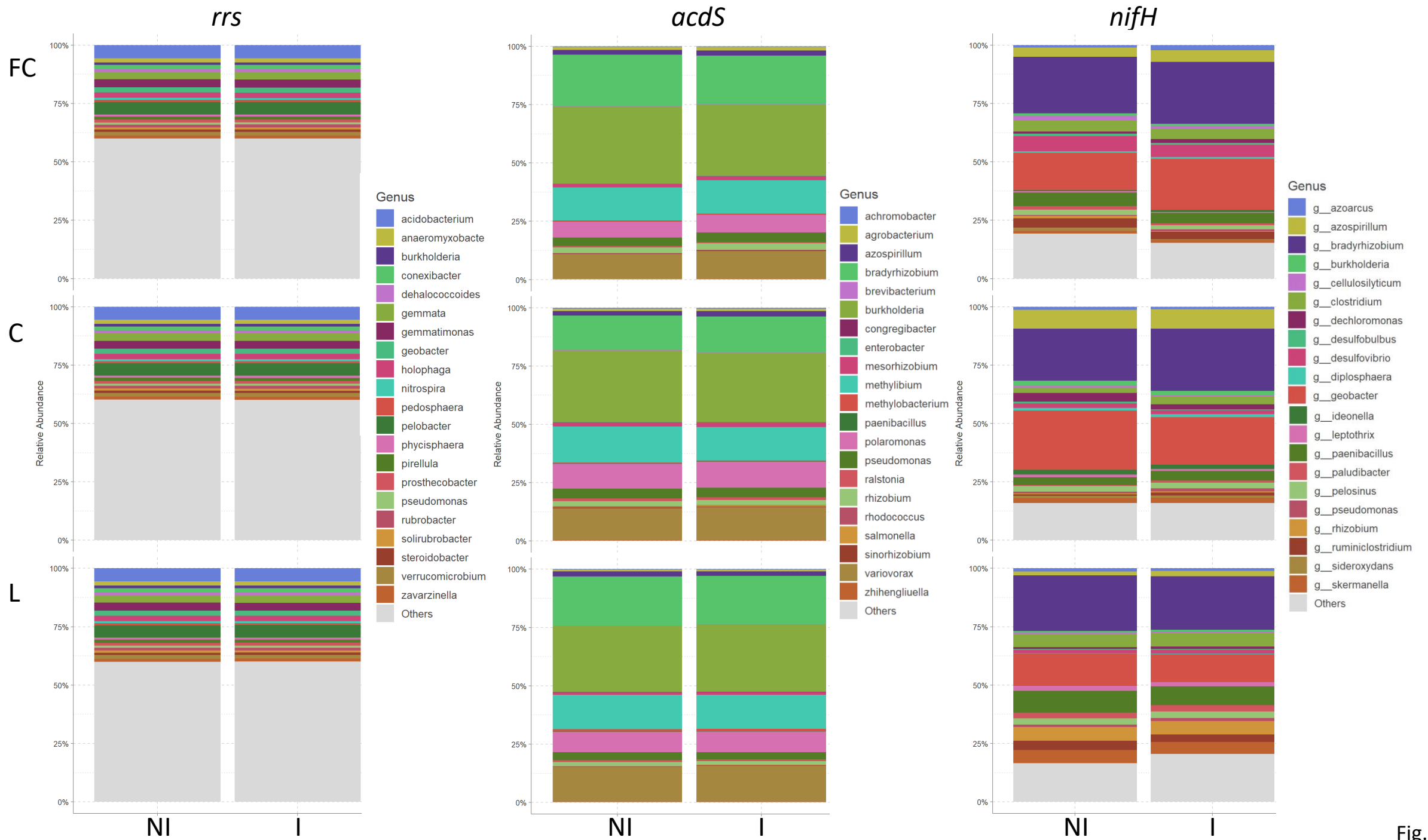

Fig. S3

Supplement: Supplementary Figure 2 — Non-metric multidimensional scaling (NMDS) of nifH (A), acdS (B), and rrs (C) data at field sites L, FC, and C. NI and I correspond, respectively, to non-inoculated and inoculated conditions. NMDS of distance matrices was based on the Bray–Curtis distance and was done using the vegan package in R. [file Data_Sheet_4.PDF]

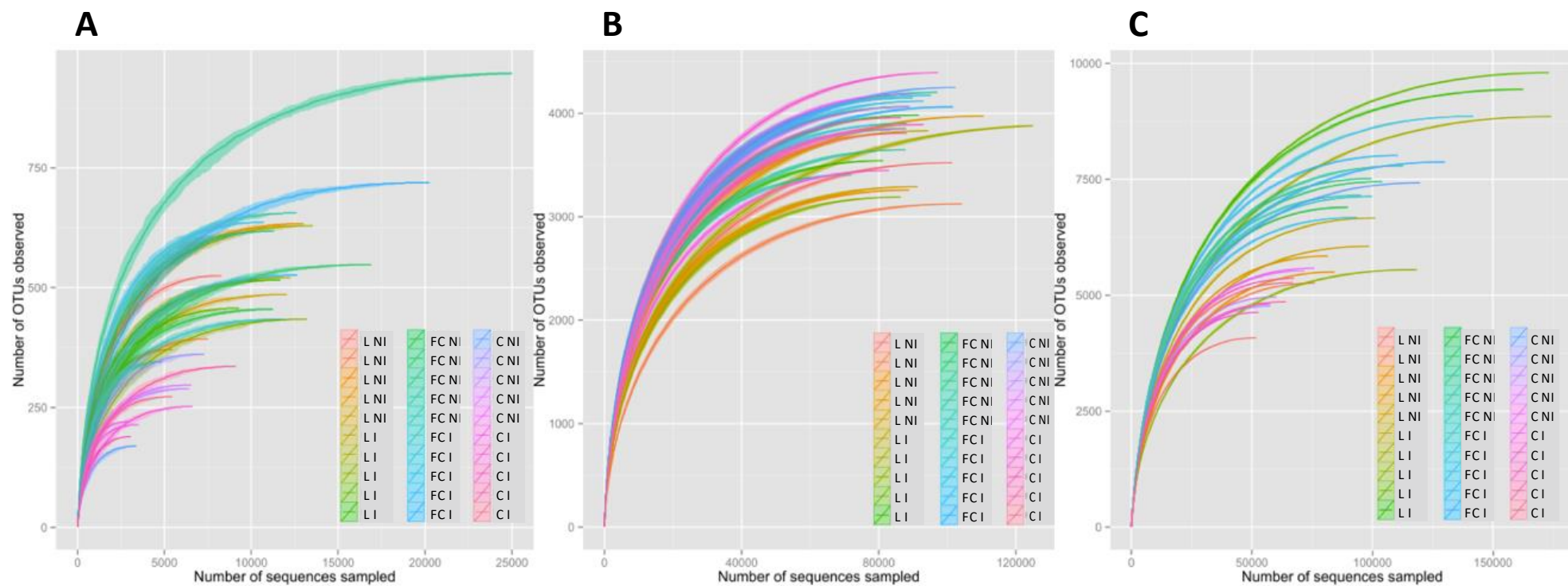

Fig S1

Supplement: Supplementary Figure 3 — Relative abundance of the most prevalent bacterial genera based on analysis of nifH, acdS and rrs data for field sites L, FC, and C (results from the five replicates were pooled). NI and I correspond, respectively, to non-inoculated and inoculated conditions. [file Data_Sheet_5.PDF]
