# Supplementary material for: Field Site-Specific Effects of an Azospirillum Seed Inoculant on Key Microbial Functional Groups in the Rhizosphere
Source: Front Microbiol. 2022 Jan 26;12:760512. doi: 10.3389/fmicb.2021.760512 (PMC8825484; doi:10.3389/fmicb.2021.760512)
Supplement: Supplementary file 4 [file Data_Sheet_1.PDF]

File S1. qPCR methods.

The reaction for *nifH* or *acdS* was implemented in 20 µl containing 4 µl of PCR-grade water, 1 µl of each primer (final concentration 0.50 µM), 10 µl of LightCycler-DNA Master SYBR Green I master mix (Roche Applied Science, Meylan, France) and 2 µl of sample DNA (5 ng). The reaction for *phlD* was carried out in 20 µl containing 1.9 µl of PCR grade water, 1 µl of each primer (final concentration 1 µM), 10 µl of LightCycler-DNA Master SYBR Green I master mix (Roche Applied Science), 0.5 mg of T4g32 protein, 3% DMSO and 2 µl of sample DNA (10 ng). PCR was done with 10 min at 95°C, followed by 50 cycles of (i) 95°C for 15 s (*nifH*), 94°C for 15 s (*acdS*), or 94°C for 30 s (*phlD*), (ii) 64°C for 15 s (*nifH*), 67°C for 15 s (*acdS*), or 67°C for 7 s (*phlD*), and (iii) and 72°C for 10 s (*nifH* and *acdS*) or 15 s (*phlD*). Melting curve calculation and Tm determination were carried out using the Tm Calling Analysis module of Light-Cycler Software v.1.5 (Roche Applied Science). Real-time PCR quantification data were converted to gene copy number per gram of lyophilized root-adhering soil, as done previously (Bouffaud et al. 2018).

Bouffaud, M.-L., Renoud, S., Dubost, A., Moënne-Loccoz, Y., and Muller, D. (2018). 1-Aminocyclopropane-1-carboxylate deaminase producers associated to maize and other *Poaceae* species. *Microbiome* 6, 114.
