# Supplementary material for: Field Site-Specific Effects of an Azospirillum Seed Inoculant on Key Microbial Functional Groups in the Rhizosphere
Source: Front Microbiol. 2022 Jan 26;12:760512. doi: 10.3389/fmicb.2021.760512 (PMC8825484; doi:10.3389/fmicb.2021.760512)
Supplement: Supplementary file 5 [file Data_Sheet_2.PDF]

TABLE S1 Physicochemical characteristics of topsoil at field sites L, FC and C

Field	L	FC	C
**Granulometry**			
Coarse sand (g/kg)	221	125	52
Fine sand (g/kg)	208	144	104
Coarse silt (g/kg)	172	118	197
Fine silt (g/kg)	257	265	544
Clay (g/kg)	142	347	103
**Acidity status**			
pH water	7.26	7.05	8.16
pH KCl	6.74	6.28	7.72
$CaCO_3$ (g/kg)	2	4	840
**Organic status and C:N ratio**			
Organic carbon (g/kg)	21.5	31.6	25.9
Organic matter (g/kg)	37.0	54.3	44.6
Total nitrogen (g/kg)	1.6	3.4	3.1
C:N ratio	13.4	9.3	8.4
**Nutriments**			
Phosphorus (g/kg)	0.135	0.036	0.040
Potassium (g/kg)	0.184	0.164	0.056
Calcium (g/kg)	2.84	4.84	9.33
Magnesium (g/kg)	0.051	0.111	0.034
**Cation-Exchange Capacity (cmol/kg)**	9.3	22.8	9.7
